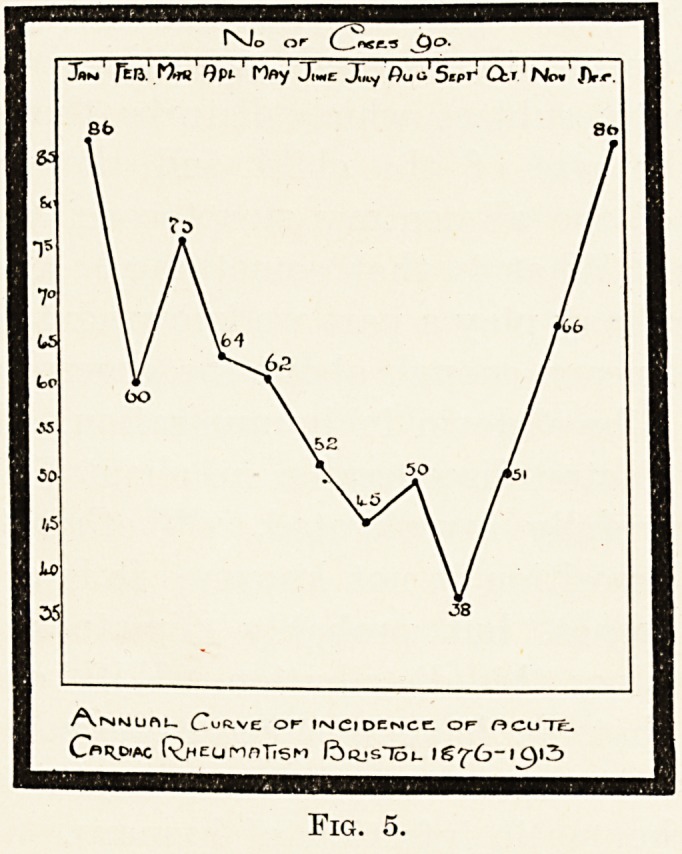# The Long Fox Memorial Lecture: The Ætiology of Cardiac Disease

**Published:** 1926

**Authors:** Carey F. Coombs

**Affiliations:** Physician (with charge of Out-patients), Bristol General Hospital;


### PLATE I.

*Early aortic syphilis.*

*Aortic syphilis in an atheromatous subject.*

*Aortic atheroma.*

[*Drawn by Miss D. Pillers.*

### The Bristol Medico-Chirurgical Journal

"*Scire est nescire, nisi id me*
*Scire alius sciret.*"

SPRING, 1926.

### THE LONG FOX MEMORIAL LECTURE:

DELIVERED IN THE UNIVERSITY OF BRISTOL AT THE MEETING OF THE MEDICO-CHIRURGICAL SOCIETY ON DECEMBER 9TH, 1925.

THOMAS CARWARDINE, Esq., M.S., *in the Chair.*

BY

CAREY F. COOMBS, M.D., F.R.C.P. Lond.,
*Physician (with charge of Out-patients), Bristol General Hospital;*

ON

### THE ÆTIOLOGY OF CARDIAC DISEASE.

I WAS never more surprised, and I think I can honestly add never more gratified, than when I heard that I had been chosen to deliver the Long Fox Lecture this year. It is true that a contemplation of the names of my predecessors, and of the lectures they have delivered, has filled me with a sense of my unfitness for so weighty a responsibility. Yet while such a responsibility is not assumed without a good many misgivings, these are outweighed by three considerations.

B

In the first place, I realise the kindly wish of my colleagues to recognise work done in Bristol, and this is a source of deep satisfaction. Let me therefore take this opportunity of thanking them for doing me so great an honour.

In the second place, it is also a pleasure to have an opportunity of paying a tribute to the memory of Dr. Long Fox. He died several years before I came to Bristol, so that I never saw him ; but his name was made familiar to me by my father, who had the greatest admiration for him. He used to come back from his visits to meetings in Bristol laden with fragments of wit and wisdom collected from his talks with Dr. Long Fox, and these he would retail to me with keen delight. Some years ago, also, he gave me a book by Dr. Long Fox, a volume on cerebral localisation, partly in print and partly in manuscript, and containing over fifty beautiful drawings of the brain. This book, which was a collection of observations made not for publication but for the pleasure of making it, was a product of that "student spirit" of which Sir Arthur Keith spoke not long ago, a spirit of ceaseless inquiry which, so far from being crushed by the routine of a heavy practice, discovers in that very routine the material on which it feeds and grows. His humility was the sign manual of the true student, and it is fitting that our school should do honour to itself, as well as to Edward Long Fox, by this commemoration of his name.

My third source of gratification at being elected to this lectureship is that it gives me an opportunity of saying with an added emphasis lent by this "brief authority" something of work which has been and is being, and will, I hope, continue to be done in the study of cardiac disease and its causation.

### PLATE II.

FIG. 1.
*Submiliary nodule of acute cardiac rheumatism from wall of left ventricle.*

FIG. 2.
*The endothelial origin of the large cells of the submiliary nodule is well seen.*

FIG. 3.
*Heart of rabbit showing a gross mitral endocarditis following inoculation with streptococcus fæcalis. Heart, cut longitudinally; mitral valve seen as dark mass at top.*

In the time at my disposal little or nothing can be said of this work except in so far as it concerns the Bristol School. Yet I must find a moment in which to acknowledge the great debt that is due, not only from myself, but also from us all, to the St. Mary's School, for it is to the men of that School that we owe much of our present knowledge of rheumatic heart disease. One of the first, if not the first, to note the relation between rheumatism and heart disease was Edward Jenner, of Berkeley, but an appreciation of the extent of that relation has only gradually grown up.

TABLE I.

*Ætiological Classification of Patients with Organic Disease of the Heart seen in*

| | *Hospital Practice.* Per cent. | *Private Practice.* Per cent. |
|---|---|---|
| Congenital .. .. .. .. | 2·0 | 2·7 |
| Rheumatic .. .. .. .. | 51·0 | 25·0 |
| Ulcerative endocarditis .. .. | 10·6 | 7·6 |
| Syphilitic .. .. .. .. | 9·0 | 4·0 |
| Cardiorenal .. .. .. .. | 7·0 | 8·6 |
| Senile .. .. .. .. .. | 10·0 | 41·0 |
| Alcoholic .. .. .. .. | 1·4 | 2·5 |
| Thyroid .. .. .. .. .. | 4·0 | 2·7 |
| Various .. .. .. .. .. | 5·0 | 5·4 |

Table I. shows how large a share of the organic heart disease that we are called upon to treat is due to rheumatic infection. My own interest in the matter, begun at St. Mary's, found plenty to feed on in Bristol, and early in my investigations into rheumatic heart disease I was lucky enough to come across the intramyocardial lesion now known as the "submiliary nodule" or the "Aschoff body" (Fig. 1). Aschoff had given an account of it[1] a few months before we found it in Bristol, but I think we may claim priority so far as a realisation of its ætiological importance is concerned. It was clear that if a lesion so distinct and striking were found constantly in rheumatic carditis, and not in other infections, we had gone a long way towards disposing of the idea, which still had some adherents, that this kind of carditis might be caused by all sorts of bacteria. Examination of hearts from persons dying of various kinds of infections showed that the submiliary nodule was a constant feature of active cardiac rheumatism, and as constantly absent from other infections. Next, it was found that in all rheumatic lesions, not only in those of the cardiac muscle, changes identical with those of the submiliary nodule were found. The essential features were those of a proliferative reaction, characterised especially by formation of giant cells, with a mild leucocytosis, on a background of fibrin. Foci of this kind are found not only in the myocardium, but also in the rheumatic valve, pericardium and joint, while the subcutaneous node, so characteristic of rheumatic infection, is only the submiliary nodule writ large. Thus we were able gradually to accumulate knowledge of the process of reaction to rheumatic infection, and to realise that the giant cells represented one phase in the reply of the vascular endothelia to the irritation inflicted on them by the rheumatic virus as it trickled through the small vessels (Fig. 2).

Obviously, however, the main interest lay in applying this test to reaction to experimental infections ; for the streptococcal view of rheumatism was, and still is, received only reluctantly and sceptically by most bacteriologists, in spite of the reasoned argument presented on its behalf by Poynton and Paine. Accordingly, material furnished by various experimenters was carefully examined, and this showed that occasionally a reaction similar to that of clinical rheumatism might follow inoculation of animals with streptococci. Yet the cardiac lesions usually produced (Fig. 3) were less like those of rheumatism than those of another disease which began to be described and discussed at about that time, *i.e.* about 1908, the disease variously named chronic ulcerative endocarditis, subacute bacterial endocarditis, and endocarditis lenta. Moreover, it proved that this kind of endocarditis is practically always excited by streptococci; that these streptococci belonged to the same cultural group as those normally found in the alimentary tract of man; and that the rheumatic streptococci also belonged to this group as much as to any.

Why, then, should these streptococci become pathogenic at all? And why should they cause sometimes one kind of cardiac lesion, sometimes another? First of all, let us see what is the essential difference between these two kinds of cardiac infection. The answer is simple; the one is a pancarditis, the other an endocarditis. As the diagram (Fig. 4) shows, the heart of rheumatic infection is attacked in all its parts at once, obviously through the coronary vessels and their ramifications; while in ulcerative endocarditis the lesion is primarily and mainly one of the inner lining of the heart, the myocardium being injured only occasionally, either by direct extension therefrom or by embolism, while the pericardium never suffers. This kind of injury, it was surmised, was inflicted on the heart by organisms in the blood passing through its cavities; a hypothesis which receives strong support from observations which Dr. Hadfield and I have recently made on the nature and distribution of the lesions found in the endocarditis of swine erysipelas. These lesions are very like those of ulcerative endocarditis in man and also those of the inoculated rabbit, while the distribution of the causal bacilli—which appear to enter the circulation from the alimentary canal—leaves no doubt that they attack the heart from within its cavities and not from the coronary circulation.

Now, transferring all this to the case of the rabbit that has been inoculated with streptococci of the

Rheumatic carditis. Ulcerative endocarditis.

Fig. 4.

*Distribution of Lesions.*

rheumatic kind, or near akin to it, we find that the conception of an infection of the endocardium from within the cardiac cavities fits the facts. This is the kind of lesion that follows introduction of a large mass of streptococci into the circulation at one blow. And that is one reason, perhaps the chief reason, why these experiments have not generally provoked a cardiac lesion which faithfully imitates that of human rheumatism; the manner in which the organisms enter the circulation, and therefore the heart, is different. We do not know exactly how the infection gets into the circulation in human rheumatism, but we may surmise that it is not in the form of a single massive dose.

If we look again for a moment at the experimental lesions we shall find that something more than injection of a large dose of organisms into the circulation is required if an endocarditis is to be set up; for if this were all, every such experiment would provoke an endocardial lesion. This is far from being the case. A majority, varying in the hands of different workers, fails to excite any cardiac lesion. Evidently it is necessary that certain conditions favouring infections should also be satisfied. And a little consideration suggests that this is true of the endocarditis lenta of man. It is very unlikely that every breach of the alimentary or bronchial mucous membranes admits streptococci that succeed in infecting the endocardium; for endocardial lesions of this kind, though commoner than we used to think, are nevertheless rare. I suppose there are not more than ten or twenty cases in a year in Bristol; yet the surface of mucous membrane that always lies exposed to the assaults of these bacteria must be at least as large as that of the ground covered by these University buildings. Now, it is ridiculous to suppose that such an area as this is not penetrated thousands of times a year by streptococci. Clearly, then, we have a parallel here with the experiments; many inoculations, but few endocardial infections. And in this case we know more of the conditions favouring endocardial infection than we do in that of the rabbits. In the first place, we know that old endocardial faults, whether developmental or acquired, invite new endocardial infections. Again, it was shown by Starling and myself, as well as by observers in France and Germany, that endocarditis lenta was unduly prevalent after the war, that the increased incidence fell wholly on ex-service men, and particularly on men of first-class physique and morale ; on men, that is to say, exposed to an ordeal of fatigue such as is rarely laid on human shoulders in these days, an experience sufficiently abnormal to alter radically the biochemistry of the body. It appears, then, that in such cases as these the environment has more power in determining whether or no the valve shall be infected than the micro-organism itself has.

Let us now return to the other kind of streptococcal infection, namely, cardiac rheumatism. That the organism is at all events similar to those of the normal alimentary tract is, I believe, admitted by everyone who supports the streptococcal hypothesis of rheumatic infection. Further, tonsillitis so often precedes an outbreak of rheumatic symptoms that it is safe to assume that the infective agent may enter in this way. Perhaps, also, it uses the lymphoid tissue of the small intestine as a means of ingress. Vining[2] has brought forward clinical evidence in support of this view, and in Price's *System of Medicine* Sir Thomas Horder[3] remarks that Dr. Mervyn Gordon "demonstrated to him an enormous quantitative increase in the Gram-positive cocci lying in the lymphoid tissue of the lower part of the ileum in two or three fatal cases of rheumatic fever." Here again, however, we must admit that if it were merely a question of an organism and a breach of mucous membrane everyone would be rheumatic. There must be other conditions, on the satisfaction of which the infection depends for its success. First among these is age.

About two-thirds of patients with cardiac rheumatism are first attacked between the ages of five and fifteen. At this age the growing child is learning immunity against his alimentary saprophytes. (Watch a little Arab on the banks of the Tigris, and you will see the same kind of thing going on. His small hand scoops up a handful of water, infected in every conceivable way, which he drinks with impunity; for it is the size of his hand that determines the size of the dose. It is like the old story of Milo and the calf; his strength grows with the burden that tests it.) But during that decade something happens to the rheumatic subject which disturbs the proportion between the size of the child and the number, or virulence, of the alimentary streptococci that he has to contain. What is that something? Of the local factors that may play a part we know next to nothing, and by no means enough about the general conditions favouring a breakdown in immunisation. There is a hereditary factor discoverable in about 50 per cent. of cases carefully investigated. Whether there is a racial predisposition is not known; it is a disease of temperate zones, but probably climate rather than race determines this distribution. With one possible exception that we have not yet explored, the lower animals do not appear to be liable to anything identical with the rheumatic infection of humans.

There is a definite seasonal variation in the incidence of rheumatic infection. The curve obtained from the Bristol mortality records of 1876–1913 (Fig.5) (examined with the invaluable help of Dr. D. S. Davies, his chief clerk, Mr. W. N. Brown, and a grant from the Colston Research Society) shows a winter elevation and a summer depression. This same series of observations[4] brought out a most interesting point: to wit, that the rheumatic rate in Bristol fell when the urban populations were, so to speak, diluted by the addition to the city of semi-rural districts. Whether it is true that rheumatism is in general a disease of urban rather than rural communities is, however, not yet proven, and I hope researches in this direction may soon be initiated on an adequate scale. It is usually regarded as a poverty disease, as witness the contrast between its prevalence in the elementary schools and its almost entire absence from the big Public

Annual Curve of Incidence of Acute Cardiac Rheumatism Bristol 1876-1913

Fig. 5.

Schools. You will notice also that it bulks twice as large in hospital as in private practice. In the Bristol investigations there was some evidence connecting this disease with overcrowding, but Coates and Thomas,[5] working in Bath, have been unable to substantiate the view that the rheumatism of childhood is a poverty disease. What they did find, however, was that the incidence was definitely influenced by elevation. The disease was more likely to occur in houses on alluvial soil, that is to say, in houses lying low and near to waterways. This agrees with Langmead's[6] experiences in London and Thomson's[7] in Birmingham. My Bristol map was rather against this view, except that it might have been interpreted as showing a high incidence in the slope of Clifton Wood overlooking the Avon. Drs. Askins and Morrison are now occupied in a very careful investigation of the homes of rheumatic children in Bristol, and we shall look forward to the outcome of their researches. Similar inquiries are in progress in other cities, partly under the auspices of the Medical Research Council, partly in reply to questions asked by a Sub-Committee of the British Medical Association, the Secretary of which, Dr. Reginald Miller,[8] himself published, some time ago, data in support of the view that in Paddington the house of the rheumatic child is a damp house.

Remarking, in passing, that our Bristol data gave no support at all to the view that rheumatism is a disease which haunts certain houses, I must now return from these rather controversial aspects to a summary of what appears established, namely that age, heredity, geography, season and perhaps an economic factor, conspire to break down the immunity of the individual against his alimentary streptococci; and that, just as this is a definite and constant combination of circumstances, so there issues from that combination a definite and stereotyped disease of the heart. That a disease which depends so much for its production on a combination of various circumstances should be separated from its neighbours by a kind of no-man's-land rather than by a sharp boundary will surprise no one; what is surprising is that time after time these conditions should so well agree as to cause a disease the histology of which is so constant and individual. And it is because we cannot yet imitate these conditions in experiments that we have so far failed to provoke, by inoculations with the appropriate streptococci, a pancarditis similar to that observed in man.

The accompanying table may serve to summarise what has been said of these streptococcal infections of the heart.

Table II.

*In man* streptococci, probably alimentary, may enter the circulation and infect the heart, either—

| Inevitably. | Accidentally. |
|---|---|
| Under certain specific conditions of age, heredity, geography, season, etc. | By finding unusual predisposing conditions, general or local. |
| They enter by the coronary vessels. | They attack from the blood circulating through the cardiac cavities. |
| The whole heart—valves, muscle and pericardium—is attacked. The histological pattern is constant and distinctive. | The endocardium is attacked first. The myocardium may be subsequently and secondarily attacked, either by direct extension or by embolism. |
| The valve is injured from within outwards. | The valve is injured from without inwards. |
| The only lesions at all resembling these rheumatic lesions in their intracardiac distribution are those of syphilis. | The lesions are like those of other forms of human ulcerative endocarditis (*e.g.* pneumococcal); the endocarditis of swine erysipelas; and most of the cardiac lesions caused by inoculation of animals with non-hæmolytic streptococci. |

Next let us consider briefly the lesions of that other pancarditic infection, cardiac syphilis; and I believe that even here, where we are dealing with micro-organisms of great vigour and individuality, we shall find that the influence of circumstances is clearly discernible. Of the 39 patients included in my series who were suffering from this disease we may exclude 2 on the ground that the syphilitic origin of the lesions was not conclusively proven. Of the remaining 37 all but 4 were men, the average age was 53, and the period between infection and onset of cardiac symptoms—so far as this was ascertained—over twenty years. The symptoms of cardio-aortic syphilis are, in fact, contemporary with those of the parasyphilides of the central nervous system. In both we have the same question to answer: why are the results of infection so tardy in declaring themselves? The spirochætes are there, in the doomed tissues, within a few days or weeks of the primary infection. It is only a partial answer to say that the survival of spirochætes in certain localities is an example of the survival of the fittest. Why should it be these tissues alone that they are able to damage? The distribution of parasyphilitic lesions within the central nervous system has been explained by saying that they are due, not to syphilis alone, but to syphilis aided by the catabolic influence of fatigue. In the distribution of the cardiac lesions, also, there is more than a hint of a similar ætiology. It is on the first part of the aorta, its valves, its coronary branches and the muscle that they feed that the brunt of the syphilitic attack falls; on parts of the cardiac apparatus, that is to say, that are also directly exposed to the stresses set up by physical toil. About 90 per cent. of my patients were men, and this agrees closely with the experience of others. This fact also appears to me to favour the view that physical stress predisposes to the evolution of spirochætal lesions in the cardio-aortic area. The ages of my patients ranged from 38 to 66 ; from an age some years anterior to that at which the vessels become grossly degenerate, well on into the normal period of decrescence. Atheroma and syphilis of the aorta may overlap each other at this period, as the pictures, made by Miss Pillers from specimens kindly lent by Dr. Hadfield, show (coloured plate).

Several years ago Drs. Waldo and Herapath[9] published an account of a case of complete heart block due to syphilis. The auriculo-ventricular bundle was destroyed throughout by what may be described as a descending degeneration, with similar but less advanced changes in the sinu-auricular node. The difference between this and the gummatous lesion usually responsible for syphilitic heart block is very like that subsisting between syphilitic myelitis and tabes dorsalis. The one is anatomical, the other physiological in distribution ; and this physiological distribution follows what may be called the restless tracts of the organ concerned. It is an illustration of the familiar ætiological principle that when two injurious agencies attack any organ or tissue simultaneously they do more hurt, or, at all events, succeed more readily in doing it, than does either of them acting alone.

The same principle also finds expression in that group of maladies that has been collectively entitled "the senile heart." In everyone a deterioration of arterial and cardiac tissue sets in somewhere about the age of 35 or 40. This deterioration, you will remember, Metchnikoff laid to the charge of the bacteria in the large intestine. If this view be true, life is one long struggle against the parasitic inhabitants of the alimentary canal; the first part of it successful, in so far as the body is capable of learning the art of immunisation, that is to say, of turning to account the *élan vital* with which it is endowed; the second half doomed to gradual declension as that vital force ebbs and leaves us more and more at the mercy of the enemy within our gates. At all events, explain it how you will, the broad fact is true: from forty onwards the cardio-vascular system is wearing out. The rate of this decline is a resultant of many factors. First comes the influence of heredity. This shows up so strongly in some cases, that it is probably the most powerful of all the agencies that determine the capacity of the circulatory pump to endure. For example, in one family whose history was reported by Dr. Lucas and myself the father died suddenly at the age of 47, and of his sons one was getting anginal pain at 39, while two others died suddenly, each at the age of 32. We made a post-mortem examination of one of these, and found in the coronary arteries and the root of the aorta atheromatous changes of the kind usually found in old people. This record is interesting, not merely because it shows how strong the influence of heredity may be, but also because it suggests that that influence may bear, in certain families, on certain particular parts of their cardio-vascular apparatus. Other examples of this are found in the familial liability to cerebral hemorrhage that is often observed; and in Dr. Herapath's discovery,[10] in two brothers, of progressive interferences with auriculo-ventricular conductivity complicating rheumatic heart disease. This is remarkable, because lesions of conductivity are rare in this disease, and when they do occur they are usually transient.

But the fate of the circulatory pump does not depend only on the kind of material which it inherits. Throughout life it is exposed to assaults of all kinds; gross mechanical stresses on the one hand, and toxic influences on the other. Among the mechanical factors it is difficult to know whether to assign a place to a life of laborious muscular effort. You will notice that the senile heart group accounts for over 40 per cent. of the private patients and only 10 per cent. of the hospital ones; yet this series included far more labouring men than that did. Too much must not be made of this antithesis, but my own experience has been that cardiac breakdown at and after sixty is the fate of the sedentary rather than of the manual worker. Nor can it be shown that a persistently rapid beat wears out the heart. The cardiac changes that ensue upon protracted thyroid intoxication are directly due to the action of toxic substances on the cardiac muscle, and not to the wearing effect of continued tachycardia. That rapid beating does not of itself cause the heart to degenerate is proved by the fact that patients with the irritable heart syndrome that we saw on so large a scale during and after the war do not develop premature myocardial decay.[11]

On the other hand, there is no denying that the heart's reserves are wasted by hyperpiesia, the continued high tension that is apparently "idiopathic." In a series of patients with cardio-sclerosis but no signs of renal disease the arterial tension was persistently high in 40 per cent. While it must be admitted that the distinction between renal hyperpiesis and essential hyperpiesia may eventually prove to be artificial, there is no doubt whatever as to the very large part played by high tension, however it may be caused, in forcing the heart into bankruptcy.

On the other hand, too much has been made of the mechanical burden laid on the senescent heart by chronic pulmonary disease. Yet of the toxic influences that may spell ruin to the myocardium one of the most potent and prevalent is the action of bacterial poisons generated by persistent respiratory infections. That this should be so is not surprising. The action on the myocardium of pneumococcus toxin in large doses is familiar, and it is only to be expected that the same poison, administered in small doses but over a long period, should work havoc of a less devastating but more permanent character. In a number of elderly patients with chronic pulmonary infections the influence of bacterial toxins on the cardiac muscle has been quite unmistakable, one of the most striking proofs of the relation being furnished by the improvement in the state of the heart which may follow an abatement of the pulmonary infection.

Another possible relation between infection and myocardial breakdown in later life is illustrated by a case which Dr. Hadfield and I are publishing.[12] A man of 49, the subject of syphilitic aortitis, died in the midst of an attack of lobar pneumonia. Post-mortem the descending branch of his left coronary artery was found to be completely closed by thrombosis and a large area of the ventricular wall had become necrotic. Gross coronary thrombosis is not very rare, and in recent years a number of papers have been written about it, especially by American physicians. (See also recent papers by Gibson[13] and McNee.[14]) In the course of a discussion on the subject Professor Francis Fraser made the very interesting suggestion that often cardiac failure in elderly men is the outcome of a series of coronary thromboses, only the grossest of which are appreciable as such clinically. There is a certain amount of anatomical evidence in support of this proposition, which, if read in conjunction with the signs of an infective process, such as fever and pericardial friction, which often appear during the establishment of a coronary obstruction, adds a wider significance to the case we are reporting. Here there is no doubt that an acute infection, pneumonia, precipitated the deposition of clot in arteries already damaged by syphilis ; and it is not improbable that many of the coronary thromboses in elderly men are the outcome of some new infection operating in a vessel whose endothelial continuity was already broken. If this is so, it seems that even in the deterioration of cardiac muscle that is the common lot of man after middle life the toxins of bacteria may play a threefold part. It is perhaps through their action on tissues whose vitality is decreasing that arteries, and therefore cardiac muscle, become old ; toxins derived from bacteria in the respiratory tract may also make a direct attack on the impoverished myocardium ; and by a similar agency the coronary vessels may become suddenly occluded, to the undoing of a heart already weary of its task.

And on that note I must make an end of this short summary. I wish it might have been rounded off with a comparison between the lesions of the greater or systemic circuit and those of the lesser or pulmonary circuit which a group of us has been studying ; but this demands a separate report. When it appears, it will, I think, support the thesis that the present address has preached, namely that diseases of the heart arise, not from single causes only, but from conspiracies of causes. It is not the seed alone that matters, but also the soil and the weather. This, the critic may say, is so obvious that there was no need to spend an hour over it. But I am sure that we do need to think of cardiac disease in terms of causes.

More and more we are being driven back on ætiology as we see more and more clearly that the hope of advance lies in prevention rather than in cure. Yet the movement towards an ætiological view of cardiac disease has scarcely begun, if we are to judge from the kind of headings that still appear in the books. The subject-matter is still classified almost wholly in terms of morbid anatomy and physiology, hardly ever in terms of ætiology. Neither in our chief systems of medicine, nor in our popular text-books, nor even in recent manuals on heart disease will you find a paragraph on rheumatic heart disease as such, the subject being dealt with in scraps under all sorts of headings, such as mitral stenosis, pericarditis and so forth. The subject of cardiac syphilis fares no better; yet how are we to look for success in treatment if we are not taught to recognise the perfectly definite clinical picture furnished by this disease? In all sincerity I hope the work we are doing in Bristol will help to bring order into this desperate chaos, and I must therefore thank you once more for giving me this opportunity of enlisting your interest in our studies.

REFERENCES.

1 Aschoff, *Brit. M. J.*, 1906, ii. 1,103.
2 Vining, *Lancet*, 1924, ii. 215.
3 1924, London (1st Edition), 287.
4 Coombs, *Lancet*, 1920, ii. 226.
5 Coates and Thomas, *Lancet*, 1925, ii. 326
6 Langmead, *Lancet*, 1911, ii. 1,133.
7 Thomson, *Brit. M. J.*, 1925, ii. 794.
8 Miller, *Brit. M. J.*, 1923, ii. 702.
9 Waldo and Herapath, *Lancet*, 1922, i. 271.
10 Reported in *Rheumatic Heart Disease.* Coombs, 108. Bristol: John Wright & Sons Ltd., 1924.
11 Grant, *Heart*, 1925, xii. 121.
12 Coombs and Hadfield, *Lancet*, 1926, i. 14.
13 Gibson, *Lancet*, 1925, ii. 1,270.
14 McNee, *Quart. J. Med.*, 1925, xix. 44.

## Figures and Tables

**Figure f1:**
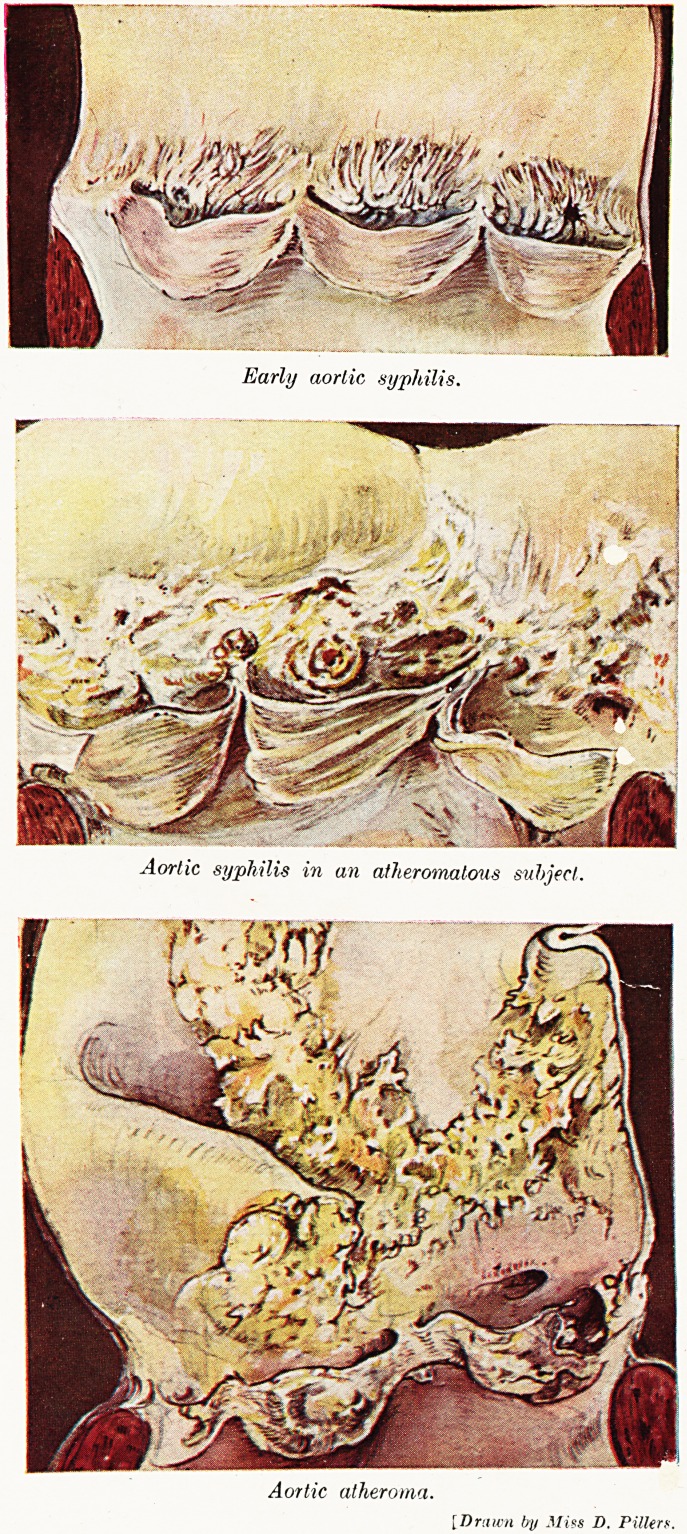


**Fig. 1. f2:**
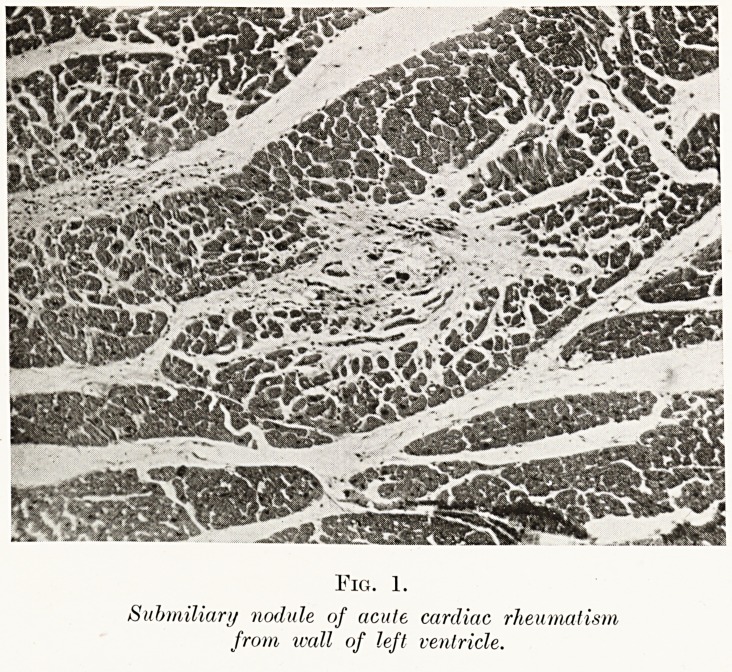


**Fig. 2. f3:**
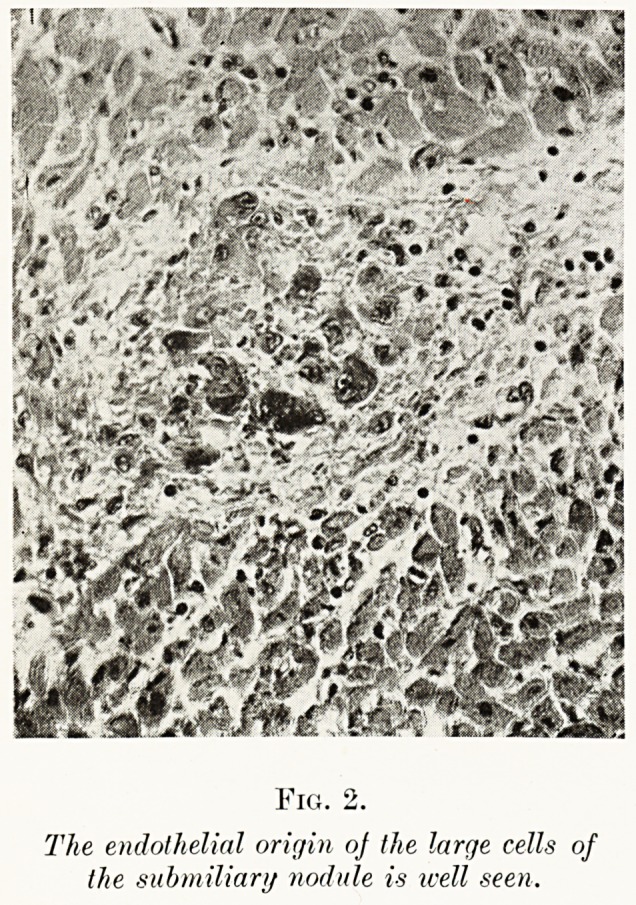


**Fig. 3. f4:**
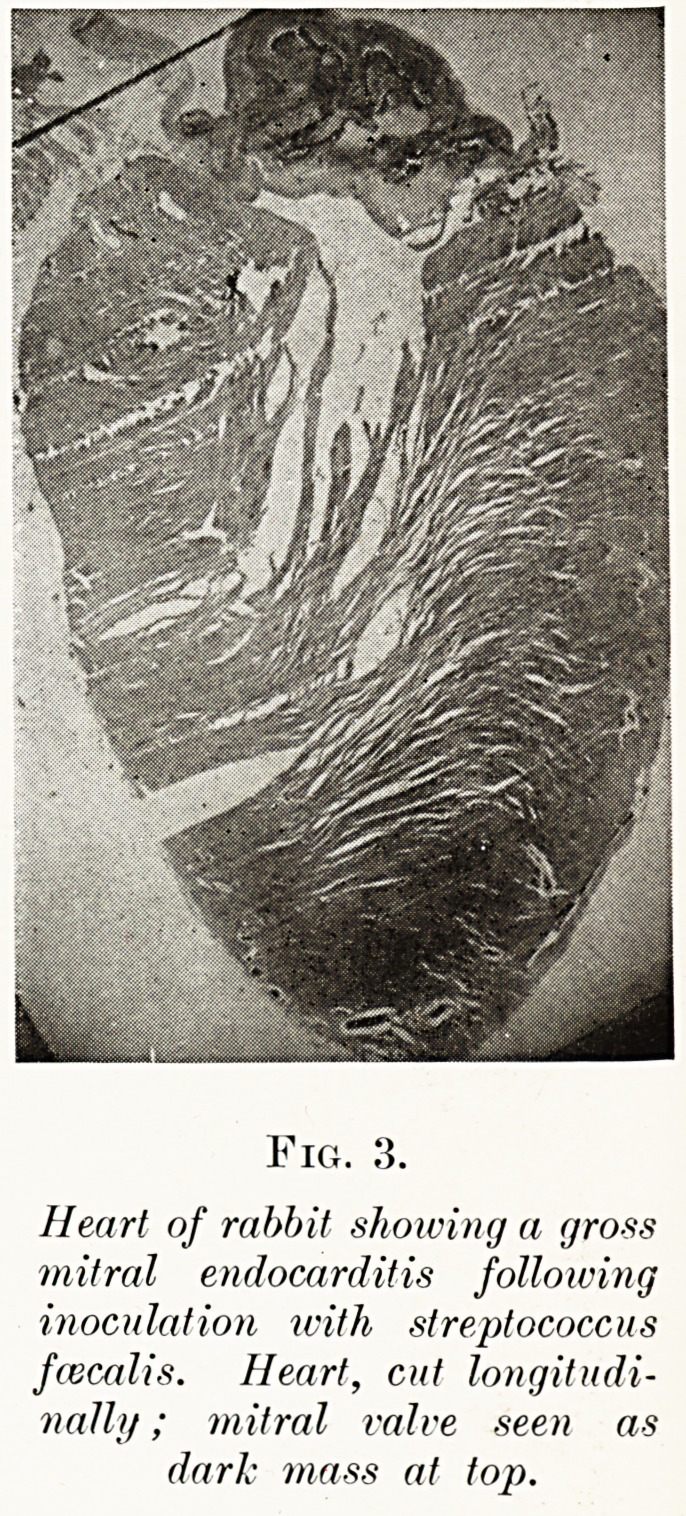


**Fig. 4. f5:**
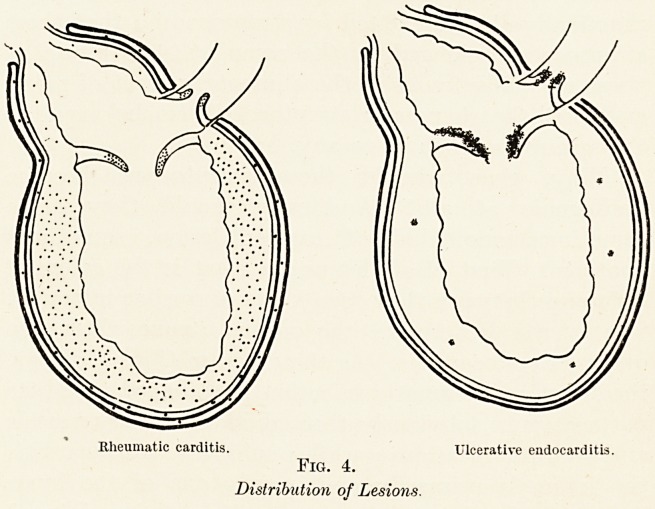


**Fig. 5. f6:**